# How Ocular Surface Microbiota Debuts in Type 2 Diabetes Mellitus

**DOI:** 10.3389/fcimb.2019.00202

**Published:** 2019-06-17

**Authors:** Siqi Li, Guoguo Yi, Hui Peng, Zhenhao Li, Shuze Chen, Huimin Zhong, Yifan Chen, Zhoucheng Wang, Qixin Deng, Min Fu

**Affiliations:** ^1^Department of Ophthalmology, Zhujiang Hospital of Southern Medical University, Guangzhou, China; ^2^School of Nursing, Hunan University of Medicine, Hunan, China

**Keywords:** ocular surface, diabetes mellitus, microbiota, flora, infection

## Abstract

High glucose represents a good environment for bacterial growth on the skin, on the ocular surface (OS) and in the tears of type 2 diabetes mellitus (T2DM) patients, affecting the conjunctival bacterial community. This study aimed to investigate the OS bacterial flora of T2DM patients and healthy subjects using 16S rRNA sequencing-based bacterial identification. Among 23 healthy subjects (CON) and 31 T2DM patients, 54 eyes were examined to investigate the OS bacterial community. Factors potentially affecting the microbial growth were controlled. Results showed the OS microbiota presented higher diversity in the T2DM group than in the CON group. Bioinformatic analysis showed a lower abundance of *Proteobacteria* and a higher abundance of *Bacteroidetes* at the phyla level as well as a significantly increased abundance of *Acinetobacter* and *Pseudomonas* at the genus level in the T2DM group. The difference in OS microbiota at taxonomic level was associated with Ocular Surface Disease Index and course of T2DM. These findings indicate the OS flora in T2DM patients is significantly different from that in healthy subjects, which may be closely associated with OS discomfort and course of T2DM.

## Introduction

Type 2 diabetes mellitus (T2DM) is a common chronic disease and may cause many ocular complications, such as dry eye and ocular infection, which significantly affects the quality of life and increase the medical burden. The prevalence of T2DM in China is approximately 11% (Shin et al., 2016). In T2DM patients, high glucose level represents a good environment for bacterial growth on the skin, on the OS and in the tears, which may alter the conjunctival bacterial community. In a majority of studies, bacterial culture is a main tool to identify the pathogenic bacteria in the OS of DM patients. The difference in conjunctival bacterial community between healthy subjects and DM patients using the culture method has revealed increases in both Gram-negative bacteria (Phillips and Tasman, 1994) and Gram-positive bacteria (Martins et al., 2004) including *Klebsiella pneumonia* (Fernandez-Rubio et al., 2010), *Gram-negative cocci* (Bilen et al., 2007), *Escherichia coli* (Adam et al., 2015), *Staphylococcus epidermidis, Staphylococcus aureus* (Zhang et al., 2014), *listeria* and *diphtheroid bacillus* (Martins et al., 2004), and some of them are pathogenic bacteria. However, culturing can only identify the specific species of bacterial flora, and the *in vitro* environment, incubation time, and other factors also affect bacterial growth during bacterial culture. High-throughput sequencing for 16S rRNA analysis can reduce the potentially confounding factors in the bacterial culture. 16S rRNA sequencing-based bacterial identification has been widely applied in investigating the intestinal canal (Musso et al., 2011), vaginal canal, oral cavity, and amniotic fluid (Wang et al., 2018) microbiota of TM patients. Furthermore, this technique is used to investigate the OS bacterial flora in patients with dry eye (Graham et al., 2007), blepharitis (Lee et al., 2012), and contact lens wearing (Shin et al., 2016) and in healthy subjects (Huang et al., 2016). However, the mechanism underlying the OS metabolic change due to the alteration of OS microbial community is still unclear. This study aimed to investigate the microbial composition in the OS of T2DM patients, thus providing evidence for further investigation of the mechanism underlying the OS metabolic change due to the alteration of OS microbiota.

## Materials and Methods

### Study Participants

This study was approved by the Ethics Committee of Zhujiang Hospital of Southern Medical University and written informed consent was obtained from all subjects. This study was conducted in accordance with the Declaration of Helsinki. A total of 54 subjects (24 males and 30 females) with a mean age of 52.98 ± 16.56 years were enrolled from Zhujiang Hospital of Southern Medical University in Guangzhou, China. The inclusion criteria were as follows: Subjects did not wear contact lenses or have any medical history of systemic diseases and OS diseases including dry eye, glaucoma, blepharitis, nasolacrimal duct obstruction or anterior segment infection, uveitis, retinal disease, or ocular trauma or transplantation. The following exclusion criteria were used: (1) patients had recent (<3 months) use of antibiotics, drugs, probiotics or fiber supplements that may affect the flora, antidiabetic drugs or weight-loss treatments; (2) subjects had anemia, gastrointestinal disorders, or chronic diseases; (3) subjects were pregnant or breast-feeding; (4) subjects had unusual dietary habits (vegetarians and vegans); and (5) individuals received eye drops treatment (antibiotics, corticosteroids, and non-steroidal anti-inflammatory drugs) within the prior 6 months. According to the diagnostic criteria of T2DM, the participants were divided into a DM group (*n* = 23) and a control (CON) group (*n* = 31). T2DM patients had no complications including diabetes-related ketoacidosis, renal failure, blindness, limb amputation, hyperglycemia related to type 1 DM or other types of DM (Table 1).

**Table 1:** Illumina sequencing and statistical data.

	**CON (*n* = 23)**	**T2DM (*n* = 31)**	***P***
Male	13	17	>0.90
Female	10	14	
Age	43.88 ± 17.43	56.68 ± 15.13	0.076
PD-whole-tree	111 ± 19.27	100.83 ± 7.67	0.105
Chao1	1997.38 ± 145.03	1949.07 ± 263.07	1
Dominance	0.06 ± 0.04	0.08 ± 0.04	0.074
Observed species	1076.29 ± 274.28	907.61 ± 99.89	0.009^*^
Shannon	6.82 ± 0.94	6.15 ± 0.74	0.04^*^
Simpson	0.94 ± 0.04	0.92 ± 0.04	0.074

*Values are presented as means ± SD. Statistical analysis was done by using the Mann–Whitney U-test*.

* *P < 0.05 vs. CON group*.

T2DM was diagnosed according to the 2017 US ADA diagnostic criteria: (1) glycosylated hemoglobin HbA1c is ≥ 6.5% and (2) fasting blood glucose (FPG) is ≥7.0 mmol/L. Fasting is defined as no caloric intake for at least 8 h. In the oral glucose tolerance test, the blood glucose is ≥11.1 mmol/L for 2 h. (3) In patients with typical hyperglycemia or hyperglycemic crisis, the random blood glucose is ≥11.1 mmol/L (2018).

### Questionnaire

All subjects were requested to filled in a questionnaire which included two parts: a baseline information section and an ocular discomfort section. The baseline information included sex, age, presence, and severity of ocular discomfort, duration of DM, history of systemic or ophthalmic medication, history of ophthalmic surgery, history of use of contact lens, and history of system diseases. The ocular surface disease index (OSDI) served as an ocular discomfort, noting the experience of the patients in prior 3 months (Pakdel et al., 2017). The severity was graded on a scale of 0 to 4: 0, no discomfort; 1, some of the time, 2, half of the time; 3, most of the time; and 4, all the time (Schiffman et al., 2000). The final score was calculated as follows: A ^*^ 25/B, where A is the sum of scores for all questions answered and B is the total number of questions answered. The higher the score, the more serious the ocular discomfort is.

### Sample Collection, DNA Extraction, PCR Amplification, 16S rRNA Sequencing, and Data Analyses

For bacterial analysis, each participant received ophthalmologic examinations at Zhujiang Hospital of Southern Medical University. Topical anesthesia was applied before collection. Subjects were asked to sit in a clean room, and the ocular specimens were collected from the upper, lower palpebral, caruncle, and fornix conjunctiva using a disposable aseptic dry cotton swab from a random eye. Another aseptic dry cotton swab containing the topical anesthetic was used as a blank control. Fifty-four samples were collected from all participants (31 DM patients and 23 CON subjects) between June 2018 and September 2018. After collection, the samples were stored at −80°C until DNA extraction.

Bacterial DNA was extracted using a DNA extraction kit (MinkaGene Bacterial DNA Kit) and the concentration and purity were measured using a NanoDrop One instrument (Thermo Fisher Scientific, MA, USA) following the manufacturer's instructions. After extraction, no bacterial DNA was found in the blank controls. Twenty milliliters of elution buffer were added to each sample and the samples were immediately stored at −20°C until Polymerase Chain Reaction (PCR) analysis.

To amplify bacterial 16S rRNAV3-V4 fragments, 12-bp barcoded primers synthesized by Invitrogen (Invitrogen, Carlsbad, CA, USA) were used. All individually processed human conjunctival DNA extractions were used as templates. The PCR mixture contained 25 μl reactions Taq (Takara Biotechnology, Dalian Co. Ltd., China), 1 μl of each primer (10 mM), and 3 μl DNA (20 ng/μl) template (final volume: 50 μl). The protocol used in the PCR was as follows: 94°C for 30 s followed by 30 cycles of denaturation at 94°C for 30 s, annealing at 52°C for 30 s, and extension at 72°C for 30 s; followed by a final extension at 72°C for 10 min. The PCR products were subjected to 1% agarose gel electrophoresis and then sequencing using Illumina Miseq (PE 300) in the MAGIGENE Genomic Institute. The PCR products were mixed in equidensity ratios according to the GeneTools Analysis Software (Version 4.03.05.0, SynGene).

After the primers were removed by preprocessing the sequence reads, QIIME (version 1.8.046.) was used for sequence reading. The criteria for QIIME quality trimming were as follows: (1) truncation of the sequence before three consecutive low-quality bases and reevaluation for length; (2) no ambiguous base calls; and (3) a minimum sequence length of 100 bp after trimming. To show the relative mean abundances of bacteria in DM patient and CON subject samples, rarefaction analysis of bacterial 16S rRNA sequences was performed based on the OTU table, and results were displayed using R software. Observed species, Chao1, Shannon, Simpson, dominance, and PD_whole_tree richness analysis were performed to show the diversity, richness, or evenness of the OS microbial communities using QIIME (V1.9.1); results were displayed in R software using the K-Sample Fisher-Pitman Permutation Test. Furthermore, to evaluate the differences in species complexity among all 54 samples, weighted, and unweighted unifrac beta diversity indices were calculated using the QIIME software. Results were displayed using the QIIME and the ggplot2 package in the R software program, and Principal Coordinate Analysis (PCoA) was performed to obtain principal coordinates and visualize complex, multidimensional data. To visualize the species abundance of the top 30 species at the phylum and genus levels in two groups, heat maps were generated, and the Bray-Curtis distance matrix was calculated. LEfSe was used to determine the significance of differences between two groups and to find specific biomarkers distinguishing them.

## Results

### 16S rRNA Gene Sequencing

Deep 16S rRNA gene sequencing analysis was specifically used to investigate the OS microbiota by culling 16S rRNA of low quality, chimera sequencing and singleton operational taxonomic units (OTUs) in the present study. The same OTUs with 97% similarity were assigned. Successful PCR amplicons of 31 DM patients and 23 CON subjects were recruited using MiSeq and QIIME, respectively, and a total of 3,130,581 sequencing reads were obtained. After removing contaminated sequences, which were annotated as chloroplasts or mitochondria (16S amplicons) and could not be annotated to the kingdom level, chimeras and singleton OTUs, 2,653,369 high-quality16S rRNA gene sequences (90.76% of the total reads) were finally obtained, resulting in an average of 49136.46 sequences. There was no significant difference in age and gender between the two groups (Table 1).

### OS Microbial Community of DM Patients and CON Subjects

**Figure 1:**
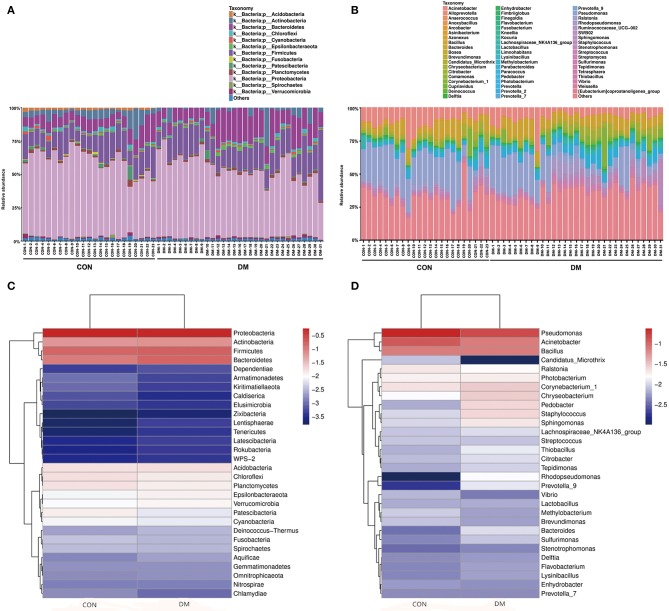
Differences in the relative abundances of phylotypes in ocular microbiota between DM patients and healthy subjects. Each phylotype (1% of average relative abundance in the groups) is indicated by a different color at the genus level. **(A)** Bacterial taxon plots at the phylum level. **(B)** Bacterial taxon plots at the genus level. **(C)** Heat map of the top 30 phyla between two groups. **(D)** Heat map of the top 30 genera between two groups.

The OS microbial community was classified into 42 phyla in the DM group and 39 phyla in the CON group, accounting for the proportion over 0.01%. However, 21 phyla were at <1% relative abundance in the DM group and 23 were at <1% relative abundance in the CON group. As shown in Figure 1, for the taxa compositions, OSs were similarly dominated by 9 major phyla including *Proteobacteria* (average: 48.86, 56.76%), *Firmicutes* (18.04, 15.66%), *Bacteroidetes* (15.83, 10.07%), *Actinobacteria* (6.43, 6.16%), *Acidobacteria* (1.79, 1.57%), *Chloroflexi* (1.42, 1.70%), *Planctomycetes* (1.32, 1.59%), *Epsilonbacteraeota* (1.16, 0.82%), and *Verrucomicrobia* (1.04, 0.89%). The top four phyla (*Proteobacteria, Firmicutes, Bacteroidetes*, and *Actinobacteria*) accounted for the majority. *Bacteroidetes* (*P* = 0.001) significantly increased and *Proteobacteria* (*P* = 0.006) markedly decreased in the DM group (Table 2). Interestingly, the OS microbiota in some subjects in two groups were dominated by a single phylum. For example, the ocular microbiota of CON-9 was enriched in *Proteobacteria*, accounting for 71.44%; in the ocular microbiota of DM-31, *Proteobacteria* accounted for only 25.97% while *Firmicutes* accounted for 50.01%.

**Table 2:** Difference of OS flora between DM group and CON group.

	**T2DM**	**CON**	***P***
**GENUS**
Staphylococcus	0.024 ± 0.060	0.010 ± 0.008	0.993
Streptococcus	0.008 ± 0.004	0.009 ± 0.004	0.546
Pseudomonas	0.139 ± 0.122	0.233 ± 0.101	0.015^*^
Acinetobacter	0.073 ± 0.056	0.111 ± 0.060	0.001^*^
Bacillus	0.072 ± 0.040	0.074 ± 0.046	0.937
Corynebacterium	0.029 ± 0.056	0.022 ± 0.042	0.882
Others	0.347 ± 0.064	0.322 ± 0.084	0.091
**PHYLUM**
Acidobacteria	0.018 ± 0.010	0.016 ± 0.006	0.813
Actinobacteria	0.064 ± 0.059	0.062 ± 0.048	0.896
Bacteroidetes	0.158 ± 0.067	0.101 ± 0.042	0.001^*^
Chloroflexi	0.014 ± 0.007	0.017 ± 0.010	0.323
Epsilonbacteraeota	0.012 ± 0.007	0.008 ± 0.004	0.122
Planctomycetes	0.013 ± 0.006	0.016 ± 0.010	0.368
Proteobacteria	0.489 ± 0.095	0.568 ± 0.095	0.006^*^
Verrucomicrobia	0.010 ± 0.005	0.009 ± 0.007	0.065
Firmicutes	0.180 ± 0.075	0.157 ± 0.049	0.252
Others	0.017 ± 0.005	0.017 ± 0.007	0.841

Values are presented as the means ± SD. Correlations are reported by Mann–Whitney U-test, and P-values are given in parentheses. Different letters in a row (

* *) indicate significant differences between the means of the different groups (P < 0.05)*.

The OS microbiota in the DM and CON groups were categorized into 46 bacterial genera, and *Pseudomonas* (13.91, 23.33%), *Acinetobacter* (7.30, 11.09%), *Bacillus* (7.18, 7.42%) and *Corynebacterium* (2.94, 2.18%) were the top four genera. However, these genera accounted for a minority and were overshadowed by a high proportion of unclassified species. In the DM group, the abundances of *Acinetobacter* (*P* = 0.015) and *Pseudomonas* (*P* = 0.001) significantly decreased (Table 2). The relative abundances of known genera and unclassified bacteria also varied among individuals. For instance, CON-19 had the lowest relative abundance (7.20%) of *Pseudomonas* but showed predominantly other bacteria (58.25%). In addition, the high relative abundance of other species at the genus level was unusual. Interestingly, lower abundance of *Acinetobacter* had a linear correlation with older age (*P* = 0.011, *r* = −0.343) (Supplementary Table 1 and Supplementary Data).

### Comparison of OS Microbial Community Diversity Between DM Patients and CON Subjects

**Figure 2:**
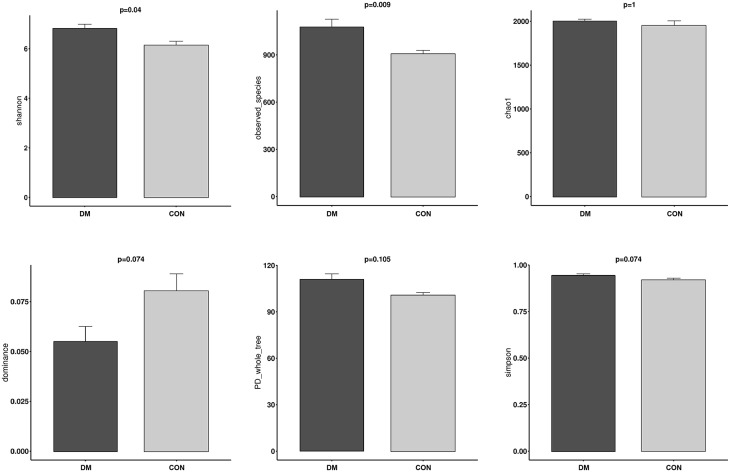
Alpha Diversity of conjunctival microbiota between DM patients and healthy subjects.

The alpha diversity of OS in the DM group was significantly higher than in the CON group based on the observed_species index (*P* = 0.009) and Shannon index (*P* = 0.04), indicating that the OS microbial community in the DM group had a greater richness and evenness than in the CON group. In contrast, the dominance index (*P* = 0.074) in the DM group represented a similar diversity in the dominant bacterial community in the CON group. The chao1 (*P* = 1), PD_whole_tree index (*P* = 0.105) and the Simpson index (*P* = 0.003) showed a similar trend with no significance between them (Figure 2 and Supplementary Data).

**Figure 3:**
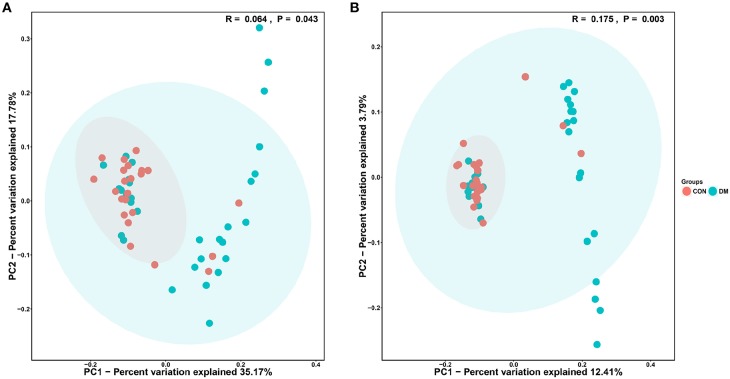
PCoA analysis of conjunctival bacterial communities in DM patients and healthy subjects. The PCoA plot was constructed using the weighted UniFrac method. The symbols represent the bacterial communities of conjunctiva from DM patients and healthy subjects. **(A)** Unweighted_Unifrac_PCoA between the DM group (blue dots) and the CON group (red dots). **(B)** Weighted_Unifrac_PCoA between the DM group (blue dots) and the CON group (red dots).

The PCoA results are shown in Figure 3, which displays the relationships among bacterial communities. This result indicated that the conjunctival microbiota in the CON group was significantly dissimilar to that in the DM group (Unweighted_Unifrac_PCoA and Weighted_unifrac; both *P* < 0.05) based on the OUT and genus profiles, which indicated the conjunctival microbiota compositions of DM patients were distinct from those of healthy subjects.

**Figure 4:**
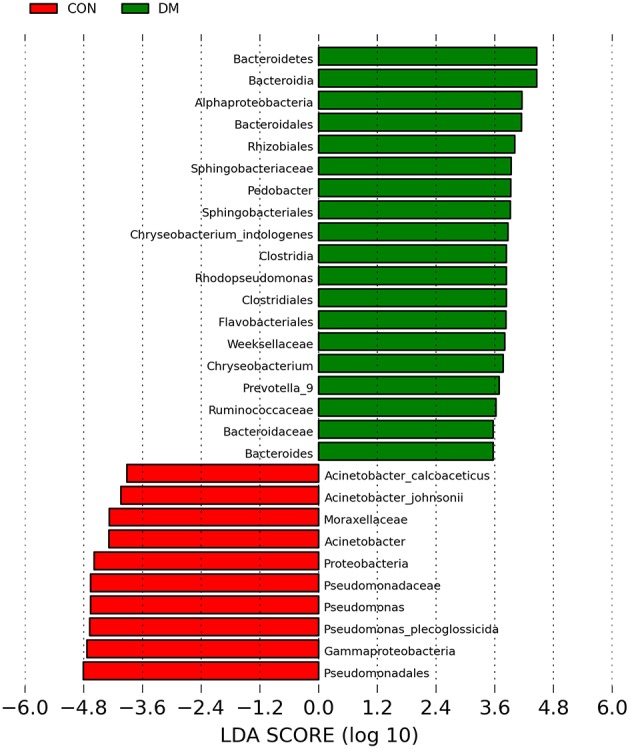
Taxa listed according to their linear discriminant analysis (LDA) values determined from comparisons between DM patients and healthy subjects using the LEfSe algorithm.

**Figure 5:**
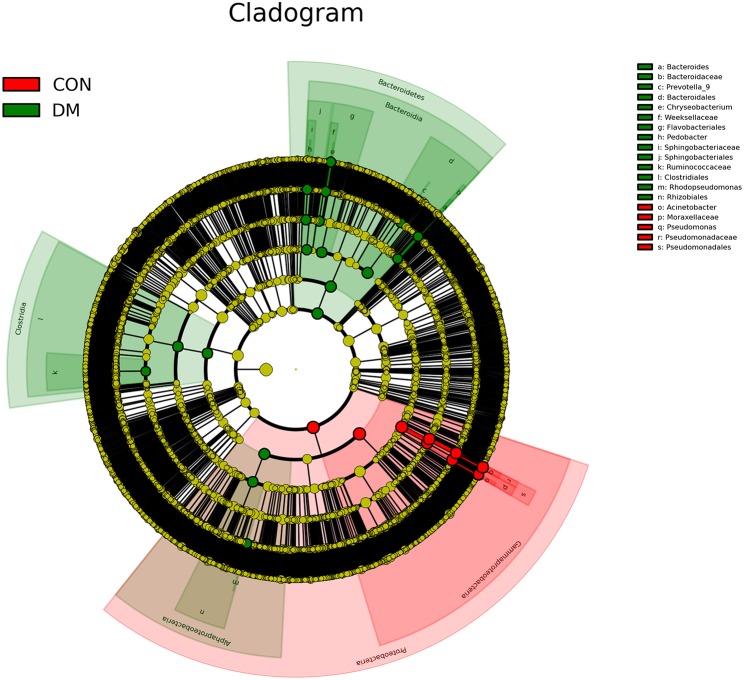
A cladogram showing differences in OS microbiota between DM patients (green blots) and CON (red blots) subjects.

To further identify the biomarkers that can differentiate DM patients from CON subjects, LEfSe analysis was performed (LDA score >3.5, *P* < 0.05). Results showed that the abundances of *Bacteroidetes, Bacteroidia, Alphaproteobacteria, Bacteroidales, Rhizobiales, Sphingobacteriaceae, Pedobacter, Sphingobacteriales, Chryseobacterium indologenes, Clostridia, Rhodopseudomonas, Clostridiales, Flavobacteriales, Weeksellaceae, Chryseobacterum, Prevotella 9, Ruminococcaceae, Bacteroidaceae*, and *Bacteroides* were significantly higher in the DM group than in the CON group (Figure 4 and Supplementary Data), and those of *Acinetobacter calcoaceticus, Acinetobacter johnsonii, Moraxellaceae, Acinetobacter, Proteobacteria, Pseudomonadaceae, Pseidomonas, Pseudomonas plecoglossicida, Gammaproteobacteria*, and *Pseudomadales* were lower in the DM group than in the CON group. A cladogram showed the differences in the OS microbiota between DM patients and CON subjects (Figure 5).

### The Relationship Among OS Microbiota Composition, Course of T2DM, and OSDI Scores

Although the OSDI scores in the DM group (5.48 ± 3.75) were similar to those in the CON group (5.15 ± 4.91, *P* = 0.506), significant correlations of OS microbiota (specific bacteria at different taxa levels) with the course of T2DM and OSDI scores were observed (Table 3). The linear regression analysis showed increases in *Acidobacteria* (*P* = 0.01, *r* = 0.457) and *Bacteroidetes* (*P* < 0.001, *r* = 0.645) and a decrease in *Proteobacteria* (*P* = 0.048, *r* = −0.358) among the top four phyla in the DM groups were associated with OSDI score and the decreases in *Acinetobacter* (*P* = 0.003, *r* = −0.518) and *Pseudomonas* (*P* = 0.037, *r* = −0.376) at genus level (Supplementary Table 3 and Supplementary Data), and the increase in *Bacteroidetes* (*P* < 0.001, *r* = 0.461) and the decrease in *Proteobacteria* (*P* = 0.001, *r* = −0.429) were related to the course of T2DM (Table 3).

**Table 3:** Relationship Between OS Microbiota and course of T2DM.

**Level**		**Course of T2DM**	
		***P***	***r***
Genus	Acinetobacter	<0.001	−0.49
	Pseudomonas	0.017	−0.323
Phylum	Bacteroidetes	<0.001	0.461
	Proteobacteria	0.001	−0.429
	Epsilonbacteraeota	0.047	0.272

*Spearman correlation analysis*.

## Conclusion

A healthy OS with a diverse microbial community plays an essential role in preventing ocular infection and dry eye complications. Available studies have shown significant changes in the microbial community of the intestine (Musso et al., 2011), vaginal canal, oral cavity, and amniotic fluid (Wang et al., 2018) by bacterial culture or 16S rRNA sequencing. The intestinal barrier function of the microbiota is weakened in DM patients (Chen et al., 2016). Thus, it is important to define the influence of DM on the ocular microbiota.

Traditional bacterial culture revealed significant differences in OS flora between healthy subjects and T2DM patients. The OS flora in the DM group included *S. aureus* (53.3%), *coagulase-negative Staphylococci* (26.7%), and *K. pneumoniae* (6.7%). In the T2DM patients, the following bacteria were identified: *S. aureus* (30%), *E. coli* (20%), *CNS* (10%), and *K. pneumoniae* (10%) (Adam et al., 2015). Karimsab and Razak (2013) investigated 100 patients with *coagulase-negative staphylococci* in the inferior palbebral conjunctiva and found the proportion of aerobic bacteria was higher in DM patients than in nondiabetic patients. Bilen et al. (2007) found that the conjunctival flora in type 1 DM patients differed from that in T2DM patients.

The traditional bacterial culture has limitations in the study of microorganism communities. Certain pathogens are difficult to grow under routine conditions, resulting in lower levels of bacterial detection when compared with the 16S rRNA sequencing or the molecular metagenomics (Zhou et al., 2014). The emerging molecular biological technique (16S rRNA sequencing) has higher accuracy in the detection of microbial community. Some studies on OS microbiota have clearly shown a higher microbial diversity by using 16S rRNA sequencing when compared with that obtained by using the traditional bacterial culture (Ozkan et al., 2017), and 16S rRNA sequencing has proven to be a more efficient tool for studying microbial communities as compared to bacterial culture.

Using high-throughput 16S rRNA sequencing, the present study provided new information on the composition of conjunctival microbiota in healthy subjects and DM patients; the average relative abundance of top 4 phyla in the ocular microbiota (*Proteobacteria, Firmicutes, Bacteroides*, and *Acinetobacter*) was similar between healthy subjects and DM patients, and similar results were noted in the top 4 genera (*Pseudomonas, Acinetobacter, Bacillus*, and *Corynebacterium*). Using the bacterial culture, Huang et al. examined 14 genera on the OS of 135 samples and found many of these were Gram-positive bacteria, which represents the cultivable microbes on OS (Huang et al., 2016). However, in our study, a total of 14 phyla and 56 genera were identified on OS in addition to unclassified organisms; these findings may not be achieved by using the traditional bacterial culture. Studies on the OS microbiota have revealed similar findings by using 16S rRNA sequencing or the metagenomics biological technique (Dong et al., 2011; Ozkan et al., 2017). *Firmicutes* and *Bacteroides* are the two main phyla in the gut bacterial community, indicating that a core human microbiome may exist in the intestine, OS, and other organs (Huse et al., 2012). According to the LEfSe analysis, among these top three phyla, *Proteobacteria* and *Acinetobacter* were less abundant on the OS of DM patients, whereas *Bacteroidetes* had a higher abundance. *Bacteroidetes* is also a major phylum in the gut bacterial community, which digests peptones and glucose to produce formic, acetic and propionic acids (Benson et al., 2010). Imbalances among the major species of the bacterial community may become detrimental to the ocular barrier.

Furthermore, the alpha diversity of DM patients was more variable than that of healthy subjects, and healthy subjects tended to show more fixed dominant flora, indicating the OS of DM patients may be unsuitable for the normal species of bacteria due to the changed metabolic environment. Pomposelli et al. (1998) found that DM patients had higher risk for infection because of higher serum glucose. The altered metabolism in DM patients may account for these changes. Hyperglycemia, inflammation, and vascular factors are also related to the changes in the ocular flora in DM patients. The hyperglycemia and high-grade systemic inflammation in DM patients may promote the growth and colonization of potential pathogens (Fernandez-Rubio et al., 2010). It has been reported that the formation and deposition of advanced glycation end products (AGEs) on the OS of DM patients may result in the destruction of extracellular matrix proteins and endothelial junctional complexes at the molecular level (Kandarakis et al., 2014). Chronic inflammatory responses at the OS lead to T cell exudation, disappearance of conjunctival epithelial goblet cells, conjunctival epithelial apoptosis and inflammatory cytokine secretion (Mantelli and Argueso, 2008). These factors can lead to increased vascular endothelial permeability, disrupt the normal barrier function of OS (Kandarakis et al., 2014) and may be responsible for the changes in ocular flora. However, some potential pathogenic bacteria remained unchanged in the DM group in the present study; these bacteria included *Staphylococcus*, the relative abundance of which was 0.024 ± 0.060 in DM group and 0.010 ± 0.008 in CON group (*P* = 0.993), and *Streptococcus*, the relative abundance of which was 0.008 ± 0.004 in DM group and 0.009 ± 0.004 in CON group (*P* = 0.546). Further study is needed to elucidate the potential mechanism. Changes in the OS flora of DM patients could ultimately lead to some adverse consequences. Studies using traditional bacterial culture showed that increased Gram-negative bacteria (especially *K. pneumoniae*) accounted for a higher risk of postoperative endophthalmitis (Phillips and Tasman, 1994; Adam et al., 2015). In studies on DM patients, significant changes in the OS flora were related to retinopathy (Martins et al., 2004; Karimsab and Razak, 2013). Our study did not further investigate the OS flora in patients with DM complications due to few patients with these complications; thus, the mechanism by which OS flora abnormalities cause diabetic ocular complications is not fully understood, and further investigation is needed. Furthermore, with more serious OS discomfort in older people (Gipson, 2013), our study did show significant decreases in *Acinetobacter* and *Pseudomonas* as well as a significant liner correlation of decreased *Acinetobacter* with age. Moreover, our study revealed a significant positive linear relationship of increased *Bacteroidetes*, decreased *Proteobacteriathe* at phylum level, and decreased *Acinetobacter, Pseudomonas* and many other genera with longer course of DM and higher OSDI scores, indicating that the changes in OS microbiota of DM patients, especially the reduced *Acinetobacter* and *Pseudomonas*, account for higher OS discomfort in these patients.

There are some limitations in this study. First, 16S rRNA sequencing is not applicable for the detection of viruses and fungi (Doan et al., 2016; Ozkan et al., 2017). Furthermore, the metagenomic approach offers a higher depth sequencing technique; therefore, further investigation of OS flora in DM patients is needed. Second, environmental exposure, occupation and age were not taken into account in the present study. Third, the diversity of microbial communities between individuals may bias the results. To date, flora transplantation treatment has been performed in the treatment of digestive diseases, achieving favorable clinical responses. Whether a similar therapy is feasible for the treatment of diabetic eye diseases is warranted to study.

## Data Availability

The raw data supporting the conclusions of this manuscript will be made available by the authors, without undue reservation, to any qualified researcher.

## Ethics Statement

The protocol was approved by the Ethics Committee of Zhujiang Hospital of Southern Medical University.

## Author Contributions

SL designed the study, collected samples, compiled the data, drafted the manuscript, and provided critical revision. GY and MF designed this study, collected samples, drafted the manuscript, and provided critical revision. HP and ZL designed this study, collected samples, and compiled the data. SC compiled the data and performed the statistical analysis and data interpretation. HZ collected samples and compiled the data. YC collected samples and drafted the manuscript. ZW and QD collected samples. MF is the guarantor of this work and, as such, has full access to all the data and takes responsibility for the integrity of the data and the accuracy of the data analysis.

### Conflict of Interest Statement

The authors declare that the research was conducted in the absence of any commercial or financial relationships that could be construed as a potential conflict of interest.
