# Supplementary material for: How Ocular Surface Microbiota Debuts in Type 2 Diabetes Mellitus
Source: Front Cell Infect Microbiol. 2019 Jun 17;9:202. doi: 10.3389/fcimb.2019.00202 (PMC6590198; doi:10.3389/fcimb.2019.00202)
Supplement: Supplementary file 1 [file Data_Sheet_1.docx]

| **Table 1-Difference of OS flora between DM group and CON group in age** | | | | | |
| --- | --- | --- | --- | --- | --- |
| **Genus** | **P** | **P (r)** | **Phylum** | **P** | **P (r)** |
| **Staphylococcus** | **0.942** | **0.181 (r=-0.185)** | **Acidobacteria** | **0.77** | **0.575 (r=0.078)** |
| **Streptococcus** | **0.981** | **0.011 (r=-0.343)** | **Actinobacteria** | **1** | **0.712 (r=1)** |
| **Pseudomonas** | **0.114** | **0.167 (r=-0.191)** | **Bacteroidetes** | **0.307** | **0.014 (r=0.307)** |
| **Acinetobacter** | **0.151** | **0.099 (r=0.227)** | **Chloroflexi** | **0.971** | **0.28 (r=0.971)** |
| **Bacillus** | **0.012^a^** | **0.98 (r=-0.004)** | **Epsilonbacteraeota** | **0.201** | **0.088 (r=0.201)** |
| **Corynebacterium** | **0.165** | **0.97 (r=-0.005)** | **Planctomycetes** | **0.228** | **0.755 (r=0.228)** |
| **Others** | **0.739** | **0.728 (r=0.048)** | **Proteobacteria** | **0.422** | **0.93 (r=0.422)** |
|  |  |  | **Verrucomicrobia** | **0.742** | **0.027 (r=0.742)** |
|  |  |  | **Firmicutes** | **0.08** | **0.214 (r=0.08)** |
|  |  |  | **Others** | **0.488** | **0.149 (r=0.488)** |
| **Correlations are reported by Jonckheere-Terpstra test and Spearman p (r), and P values are given in parentheses. Different letters in a row (a) indicate significant differences between the means of the different groups (P＜0.05).** | | | | | |

| **Table 2-Difference of OS flora between DM group and CON group in different genders** | | | |
| --- | --- | --- | --- |
| **Genus** | **P** | **Phylum** | **P** |
| **Staphylococcus** | **0.265** | **Acidobacteria** | **0.676** |
| **Streptococcus** | **0.862** | **Actinobacteria** | **0.931** |
| **Pseudomonas** | **0.198** | **Bacteroidetes** | **0.21** |
| **Acinetobacter** | **0.243** | **Chloroflexi** | **0.102** |
| **Bacillus** | **0.986** | **Epsilonbacteraeota** | **0.986** |
| **Corynebacterium** | **0.347** | **Planctomycetes** | **0.198** |
| **Others** | **0.192** | **Proteobacteria** | **0.454** |
|  |  | **Verrucomicrobia** | **0.394** |
|  |  | **Firmicutes** | **0.566** |
| **Correlations are reported by Mann-Whitney U test, and P values are given in parentheses. Statistical significance was set at a P value of ＜0.05** | | | |

| **Table 3—The Relationship Between Ocular Surface Microbiota Composition and OSDI scores** | | | | | | | |
| --- | --- | --- | --- | --- | --- | --- | --- |
| Level |  | DM | |  | Con | |  |
|  |  | P | r |  | P | r |  |
| genus | Acinetobacter | 0.003 | -0.518 | Bacteroides | 0.007 | -0.547 |  |
|  | Pseudomonas | 0.037 | -0.376 | Lysinibacillus | 0.044 | -0.424 |  |
|  | Chryseobacterium | 0.033 | 0.384 |  |  |  |  |
|  | Pedobacter | 0.011 | 0.452 |  |  |  |  |
|  | Thiobacillus | 0.001 | 0.583 |  |  |  |  |
|  | Rhodopseudomonas | 0.008 | 0.464 |  |  |  |  |
|  | Bacteroides | 0.001 | 0.564 |  |  |  |  |
|  | Sulfurimonas | 0.001 | 0.564 |  |  |  |  |
|  | Vibrio | 0.012 | -0.445 |  |  |  |  |
|  | Delftia | 0.021 | 0.414 |  |  |  |  |
|  | Prevotella_7 | 0.012 | 0.446 |  |  |  |  |
|  | Comamonas | 0.009 | 0.461 |  |  |  |  |
|  | Alloprevotella | 0.001 | 0.547 |  |  |  |  |
|  | Stenotrophomonas | 0.019 | 0.42 |  |  |  |  |
|  | Fusobacterium | 0.003 | 0.52 |  |  |  |  |
|  | Prevotella_2 | 0.011 | 0.453 |  |  |  |  |
|  | Bosea | 0.004 | 0.503 |  |  |  |  |
|  | Parabacteroides | <0.001 | 0.646 |  |  |  |  |
|  | Limnohabitans | 0.007 | 0.478 |  |  |  |  |
|  | Azonexus | 0.017 | 0.426 |  |  |  |  |
|  | Ruminococcaceae_UCG-002 | 0.001 | 0.583 |  |  |  |  |
|  | [Eubacterium]coprostanoligenes_group | <0.001 | 0.617 |  |  |  |  |
|  | Finegoldia | 0.039 | -0.372 |  |  |  |  |
|  | Tetrasphaera | 0.02 | -0.415 |  |  |  |  |
|  | Candidatus_Microthrix | 0.026 | -0.4 |  |  |  |  |
|  | Others | <0.001 | 0.679 |  |  |  |  |
| phylum | k__Bacteria;p__Acidobacteria | 0.01 | 0.457 | —— |  |  |  |
|  | k__Bacteria;p__Bacteroidetes | <0.001 | 0.645 |  |  |  |  |
|  | k__Bacteria;p__Epsilonbacteraeota | <0.001 | 0.711 |  |  |  |  |
|  | k__Bacteria;p__Proteobacteria | 0.048 | -0.358 |  |  |  |  |
|  | k__Bacteria;p__Verrucomicrobia | 0.031 | 0.388 |  |  |  |  |
|  | k__Bacteria;p__Fusobacteria | 0.014 | 0.436 |  |  |  |  |
| Correlations are reported by Spearman p (r), and P values are given in parentheses. Statistical significance was set at a P value of ＜0.05 | | | | | | | |
